# Functional and evolutionary significance of the unique lime-secreting hydathodes and amphistomatic leaves in *Saxifraga* (Saxifragaceae)

**DOI:** 10.1007/s00425-026-04999-9

**Published:** 2026-04-20

**Authors:** Natalia Tkach, Emmily Richter, Martin Röser

**Affiliations:** Institute of Biology, Geobotany and Botanical Garden, Halle (Saale), Martin Luther University Halle-Wittenberg, Am Kirchtor 1, 06108 Halle (Saale), Germany

**Keywords:** Alpine plants, Amphistomatic, Calcium carbonate, Epithem, Guttation, Hydathodes, Key innovation, Leaf vascularization, *Ligulatae*, Lime secretion, *Porphyrion*, Saxifrages, Stomata

## Abstract

**Main conclusion:**

The among angiosperms unique ability of many saxifrages to produce calcareous leaf incrustations through lime-secreting hydathodes is a complex anatomical and physiological syndrome that evolved only once in *Saxifraga* phylogeny

**Abstract:**

The genus *Saxifraga* comprises about 480 species primarily found in the mountains of the Northern Hemisphere. About 24% of these species have lime-encrusted leaves caused by unique lime-secreting glands, which contain epithem hydathodes that are connected to the xylem. These glands release guttation fluid presumably containing dissolved calcium hydrogen carbonate. We examined their structure in 81 representative species and subspecies using light and scanning electron microscopy (SEM). Lime incrustations are confined to two of the fifteen *Saxifraga* sections, which typically inhabit calcareous or base-rich substrates. Thus, the lime secretion may contribute to regulating internal Ca^2^⁺ concentrations, among other functions. Phylogenetic analysis of the entire genus *Saxifraga* shows that the ability to produce lime incrustations evolved once in the last common ancestor (LCA) of the sections *Ligulatae* and *Porphyrion*. However, this ability was subsequently lost in two lineages: sections *Gymnopera* and *Trachyphyllum*. The peculiar sunken hydathodes on the adaxial leaf surface and their association with camptodromous leaf vascularization support this scenario because they suggest that lime secretion is a complex anatomical and physiological syndrome that is unlikely to have evolved twice. While this syndrome plays a role in ecological adaptation and biogeography, it does not appear to be a classic evolutionary key innovation. Most species also have amphistomatic leaves with notable interspecific differences in stomatal arrangement related to hydathode arrangement. Amphistomaty is believed to enhance photosynthetic rates, which is consistent with these saxifrages’ adaptation to sunlit, high-elevation environments and their drought-tolerant, partly succulent, xerophytic growth forms.

**Supplementary Information:**

The online version contains supplementary material available at 10.1007/s00425-026-04999-9.

## Introduction

Terrestrial vascular plants typically have stomata on the underside of their leaves (hypostomatic leaves) for gas exchange. This arrangement prevents unnecessary water loss from exposure to direct sunlight and is characteristic of plants with bifacial leaves that grow in mesic environments (Parkhurst 1978; Becraft 1999; Liu et al. 2024). Examples include trees and shrubs in temperate climates, such as *Abies*, *Betula*, *Carpinus*, *Fagus*, *Ilex*, *Pinus*, *Populus*, *Pyrus*, *Quercus* and *Thuja* species, but also herbs like *Trollius*, as well as plants from shaded habitats, e.g. the forest understory, such as *Asperula*, *Begonia* and *Glechoma* species. Other arrangements of stomata can also be found in plant species that are particularly specialized in terms of their habitat and ecology (Napp-Zinn 1974–1988; Metcalfe and Chalk 1979). Aquatic plants which have floating leaves, sometimes in addition to submersed ones, such as species of Nymphaeaceae, *Callitriche*, *Hottonia*, *Lemna* (thalloid stem-leaf units), *Marsilea*, *Myriophyllum* (emergent leaves), *Nasturtium*, *Nelumbo*, *Nymphoides*, *Polygonum*, *Potamogeton*, *Ranunculus*, *Sagittaria* and *Trapa* usually have stomata on the upper side of the leaf above the water surface (epistomatic leaves), which enables gas exchange with the atmosphere (Björn et al. 2022; Liu et al. 2023). However, submersed leaves of the same species, if present, usually have no stomata, though there are exceptions (Evert 2006). Some plant species that grow under atmospheric conditions also have epistomatic leaves, including a few species of Cupressaceae, Ericaceae, Fabaceae, Myrtaceae, Onagraceae, Pinaceae, Proteaceae, Violaceae and many Poaceae (Metcalfe and Chalk 1950, 1979).

Stomata can also occur on both sides of leaves (amphistomaty). This is seen on the surfaces of unifacial and equifacial (isobilateral) leaves, which are found in many monocotyledons, including Asparagaceae, Iridaceae, Juncaceae, and Sparganiaceae. Amphistomatic leaves are especially characteristic of grasses (Poaceae) with the C_4_ photosynthetic pathway. They are also found in Araucariaceae, Ginkgoaceae, Pinaceae and other gymnosperms as well as in many dicots, including Brassicaceae, Plantaginaceae and Solanaceae (De Bary 1877; Troll 1939; Metcalfe and Chalk 1950; Becraft 1999). The leaf surfaces of these plants are sometimes oriented perpendicular to the incident light. This occurs in xerophytic trees and shrubs, such as *Eucalyptus*, other Myrtaceae as well as in *Nerium oleander* and herbaceous plants as frequently occurring in monocotyledons. The amphistomatic arrangement ensures that both sides of leaves spatially orientated in this way can effectively participate in photosynthesis. Amphistomatic leaves are predominantly found in plants that grow in open, sun-exposed environments, such as grasslands, deserts, and high-altitude ecosystems (Mott et al. 1982; Jordan et al. 2014; Liu et al. 2024). Wagner (1892) first noticed this characteristic in the flora of the Alps, reporting that only 15% of the alpine species he investigated had no stomata on the upper side of their leaves. The larger leaf surface area available for CO₂ uptake enhances gas exchange efficiency, which can significantly increase photosynthetic rates under high light intensity (Parkhurst 1978; Muir et al. 2025). Under these conditions, diffusion of CO₂ through a single epidermis can be the limiting factor for photosynthesis. Amphistomatic leaves mitigate this limitation by allowing CO₂ to enter from both surfaces. This reduces diffusion resistance and promotes a more uniform internal CO₂ concentration in the chlorenchyma (Drake et al. 2019).

However, having stomata on both leaf surfaces increases the potential for transpirational water loss. To counteract this, amphistomatic leaves often exhibit additional xeromorphic features such as thicker cuticles, smaller, sunken stomata, and a higher palisade-to-spongy parenchyma ratio. These features collectively reduce transpiration rates (Taiz et al. 2023). Additionally, some species regulate stomatal aperture asymmetrically by keeping adaxial stomata of the leaf partially closed under high vapor pressure deficits while maintaining abaxial stomatal conductance to sustain photosynthesis.

Another structural feature of the leaf epidermis that is associated with the stomata and plays a role in maintaining the plant’s water balance are hydathodes. This term was coined by Haberlandt (1894). De Bary (1877) originally described these structures as “Wasserspalten, -poren” (water stomata, water pores). Unlike active hydathodes, such as trichomes and glands, they are mostly passive water-excreting organs. Hydathodes are widespread in plants. The hydathodes are often arranged along the margins of leaves, such as in species of *Arabidopsis*, *Caladium*, *Colocasia* and other aroids and *Tropaeolum*. They are frequently found in serrated leaves, such as in *Alchemilla*, *Arabidopsis*, *Fuchsia* and *Potentilla*, on the upper side of the teeth or at the tip of the leaf blade, such as in species of *Aconitum*, *Corylus*, *Doronicum*, *Escallonia*, *Heuchera*, *Mitella*, *Platanus*, *Primula*, *Sambucus*, *Saxifraga*, *Valeriana*, many Apiaceae and Poaceae, or on the leaf blades such as in *Crassula* and *Ficus* (De Bary 1877; Singh 2014; Cerutti et al. 2019; Jauneau et al. 2020). The presence of hydathodes on leaves is often recognized by the small water droplets found on them in the morning. These droplets have not evaporated under the humid and often cool conditions of the night.

Although transpiration through stomata is the primary route of water loss, guttation, i.e. the excretion of liquid water, offers an alternative mechanism for water release, particularly under certain environmental conditions such as excessively high soil moisture, high humidity, or low transpiration rates. When the amount of water absorbed by the roots exceeds the amount of water lost through transpiration, positive root pressure pushes xylem sap upward and out through the hydathodes, leading to guttation (Cerrutti et al. 2019). Stomata and hydathodes are anatomically similar in that they both have guard cells. It is believed that hydathodes originated from stomata in some form, and the two are developmentally and physiologically related (Cerutti et al. 2019; Torii 2021). Although they both originate from epidermal protodermal cells, hydathodes differentiate into structures that are mostly permanently open and lack the turgor-based regulatory capability of true stomata (Pillitteri et al. 2008; Pillitteri and Dong 2013; Michavila et al. 2021; Torii 2021). In some cases, however, the opening of hydathode pores has been found to respond to environmental cues, such as light or the hormone abscisic acid, in a manner similar to stomata. Nevertheless, they appear incapable of complete closure (Bellenot et al. 2022). Therefore, they are a specialized modification of the stomatal apparatus adapted for hydraulic rather than gaseous exchange.

Hydathodes typically comprise three main components: a water pore, a subepidermal chamber and the epithem tissue (e.g., De Bary 1877; Haberlandt 1894; Belin-DePoux 1969; Fahn 1979; Evert 2006). The epithem is a specialized parenchymatous tissue with abundant intercellular spaces. It connects directly to the xylem terminations of minor vascular bundles, providing a continuous pathway for the movement of water and solutes. The exuded liquid contains water and various dissolved substances, including mineral nutrients, sugars, and amino acids (Cerutti et al. 2019; Routaboul et al. 2024).

Our study group, the genus *Saxifraga*, is a characteristic component of high-mountain vegetation in the Northern Hemisphere, extending northward into the Arctic and southward across the Andes to the Cono Sur region of South America. The genus comprises approximately 480 species, which are taxonomically arranged into 15 sections. Three of these sections contain several additional subgroups that are treated as subsections (Tkach et al. 2015, 2025; Carruthers et al. 2024). While many species in this genus require ample sunlight and are adapted to open vegetation, some inhabit the understory of forests and are adapted to shady, humid habitats (Webb and Gornall 1989).

**Fig. 1:**
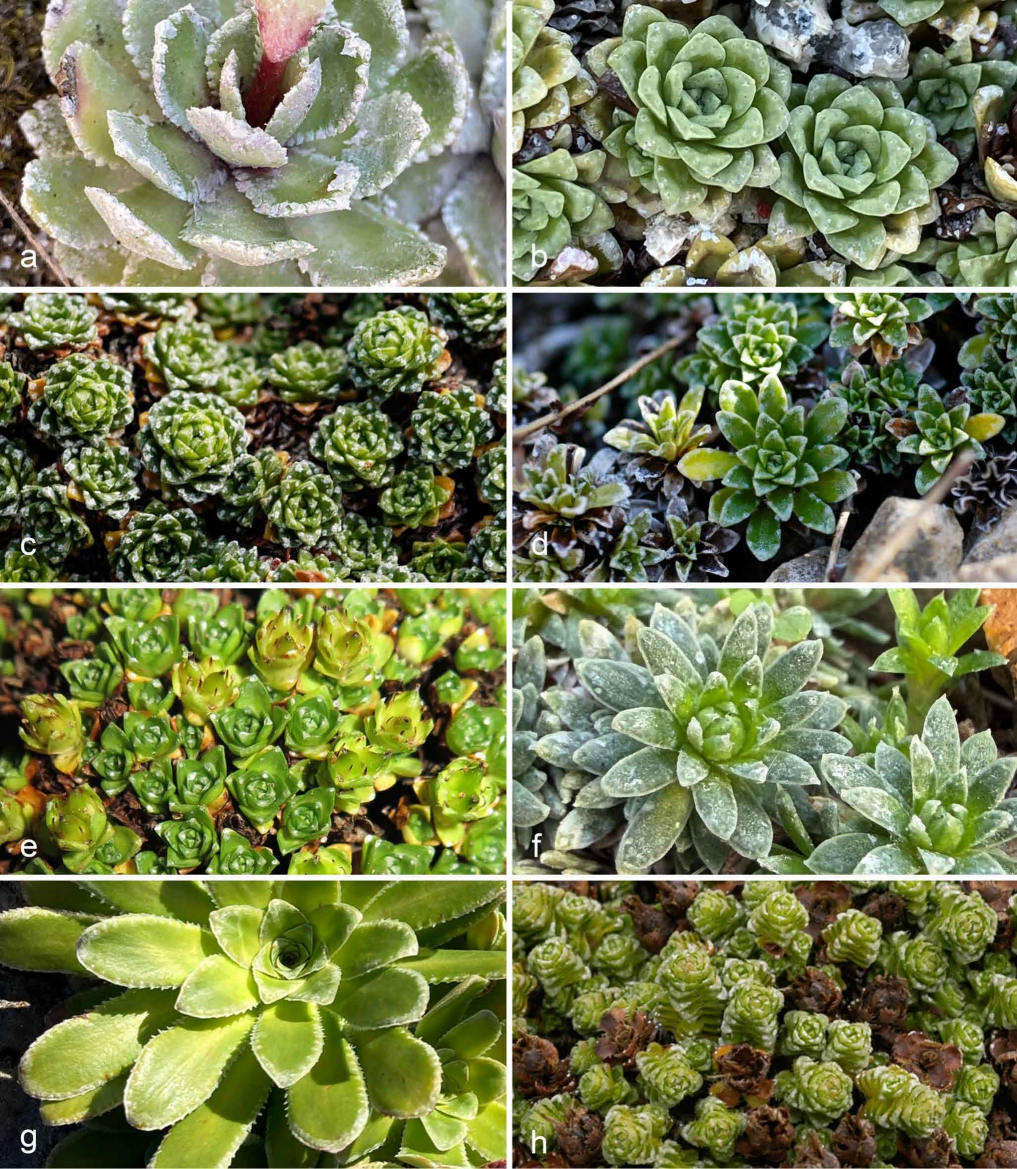
Lime-encrusted leaves and location of hydathode pits on the leaves of *Saxifraga* species from sections *Ligulatae* (**a**), sect. *Porphyrion* subsect. *Kabschia* (**b–f**), subsect. *Mutatae* (**g**) and subsect. *Oppositifoliae* (**h**). **a** *S. paniculata*; **b** *S. andersonii*; **c** *S. iranica*; **d** *S. marginata* var. *bubakii*; **e** *S. nana*; **f** *S. sempervivum*; **g** *S. mutata* subsp. *demissa*; **h** *S. oppositifolia* subsp. *latina*

**Fig. 2:**
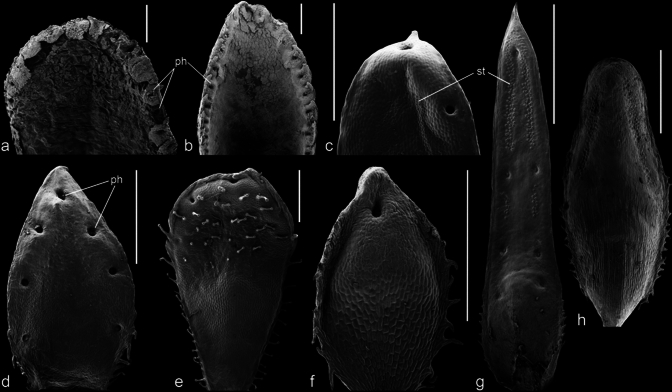
Scanning electron microscopy (SEM) preparations of adaxial (**a–g**) and abaxial leaf surfaces (**h**) of *Saxifraga* sect. *Ligulatae* (**a–b**) and sect. *Porphyrion* subsect. *Kabschia* (**c–h**). **a** *S. callosa* and **b** *S. hostii* with lime-encrusted leaves and many hydathode pits along the leaf margin; **c** *S. matta-florida* with two pits and stomata arranged in a horseshoe-shaped area; **d** *S. scardica* with five pits and stomata arranged in an arrowhead-shaped area; **e** *S. spruneri* with four pits and stomata arranged in between the multiseriate glandular hairs; **f** *S. tombeanensis* with a single apical pit and a small area with stomata distal to it; **g** *S. burseri* with seven pits and stomata arranged as narrow bands parallel to the leaf margin; **h** *S. diapensioides* with stomata arranged as an inverted V. Lime incrustations in **c–h** removed by treatment with citric acid. Scale bars represent 1 mm. *ph* pit with hydathode(s); *st* leaf area with stomata

The leaves of saxifrages have anomocytic stomata, meaning they lack subsidiary cells. They have been found to exhibit different arrangements, either hypostomatic or amphistomatic (Moreau 1984; Andrei and Paraschivoiu 2008). Additionally, the leaves usually have hydathodes in a variable number and position depending on the species. They are found on the adaxial (upper) side, along the margin, or at the tip of the leaf lamina (Unger 1836, 1861; Volkens 1883; Kurt 1930; Schmid 1930; Hofmeister 1939). These hydathodes have attracted botanical interest due to their ability to deposit calcium carbonate (CaCO₃), which Unger (1836, 1861) termed “Kohlensaurer Kalk”, and, to a minor extent, magnesium carbonate and iron salts. This lime secretion appears to be unique among angiosperms. The hydathodes produce white or silvery incrustations around their pores or along the leaf margins (Fig. 1). These incrustations are often visible as flaking scales. Sometimes, crystals can cover the entire leaf (Fig. 2a, b). Many *Saxifraga* species are calcicoles, meaning they grow on limestone rocks or in calcium-rich soils. Therefore, they prefer or tolerate high-calcium environments. These species can supposedly avoid toxic calcium accumulation in mesophyll cells by excreting excess calcium through hydathodes (Bothe 2015; Islam and Kawasaki 2015; Fehlauer et al. 2022a), as has also been found with other harmful chemicals in xylem sap (Singh 2014; Cerutti et al. 2019; Michavila et al. 2021). Using mass spectrometry and micro X-ray fluorescence spectroscopy for plant tissues, evidence was obtained of a different location of several of the otherwise colocalized chemical elements (Al, Ca, Fe, Mg, etc.) around the hydathodes of *S. paniculata* (Fehlauer et al. 2022b). This may be a prerequisite for the selective extrusion of certain elements.

Interestingly, the ability to secrete calcium-rich solutions is also occasionally present in calcifuge species belonging to calcicolous lineages. For example, in the Alps, the silicophilous *S. cotyledon* (Webb and Gornall 1989) exhibits this ability, as confirmed by personal observations (M.R., N.T.). This species is closely related to the calcicolous species *S. hostii* and *S. paniculata*. This suggests that the calcium-releasing mechanism evolved in connection with inhabiting calcareous soils but was retained when the substrate changed to low- or almost calcium-free soils. Interestingly, *S. paniculata* also forms calcareous crusts when growing on siliceous soil, albeit to a lesser extent (Webb and Gornall 1989; M.R., N.T., personal observations). All of these species belong to the same taxonomic group, i.e., the section *Ligulatae*. Similar observations were made in sect. *Porphyrion*. Most of its species grow on limestone rocks and have lime-encrusted leaves. However, several species grow on calcium-poor substrates and lack lime incrustations. They also have pits with hydathodes, hereafter termed “hydathode pits”, as their calciphilous relatives. Examples include the silicophilous *S. juniperifolia*, *S. lilacina*, *and S. pulvinaria* (Horný et al. 1986; Webb and Gornall 1989).

Previous authors have already noted this restriction to certain infrageneric groupings of *Saxifraga*, while using the names from earlier classifications (De Bary 1877; Engler and Irmscher 1916, 1919; Kurt 1930; Kaplan 1995; Conti et al. 1999; Zhang et al. 2015; Zhmylev and Kovalenko 2023). The leaf crusts of both sections, *Ligulatae* and *Porphyrion*, also appear to differ chemically. The leaf crusts of the five sampled *Porphyrion* species contained the rare calcium carbonate polymorph vaterite. By contrast, the six tested *Ligulatae* species only had calcite in their crusts (Wightman et al. 2018).

The morphological, anatomical, and histological structure of these hydathodes, as well as their development and physiology, has been analyzed in several studies conducted over the past two centuries (Unger 1836). However, these studies were mostly conducted on exemplary species (Waldner 1878; Gardiner 1881; Lazniewski 1896; Hayek 1905; Galløe 1910; Kurt 1930; Schmidt 1930; Cutler and Gregory 1998; Zhang et al. 2015; Wightman et al. 2017, 2018; Michavila et al. 2021). De Bary (1877) first reported differences in the arrangement and structure of saxifrage leaf hydathodes, noting the presence of one to four water pores on the bottom of the pits or cavites in the adaxial leaf surface. These pits are characteristic of lime-secreting saxifrages (Webb and Gornall 1989; Zhang et al. 2015; Wightman et al. 2017), whereas the water-secreting hydathodes of other saxifrages lack such pits. Galløe (1910) first described this characteristic.

**Fig. 3:**
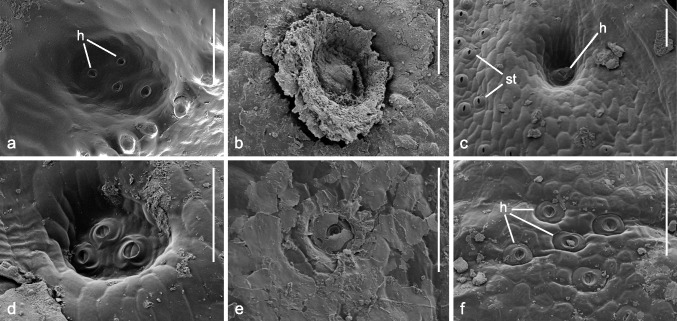
Scanning electron microscopy (SEM) preparations with details of hydathode pits on the adaxial leaf surface of *Saxifraga* sect. *Ligulatae* (**a**), sect. *Porphyrion* subsect. *Kabschia* (**b–d**), subsect. *Florulentae* (**e**) and sect. *Mutatae* (**f**). **a** *S. lingulata*; **b** *S. iranica*; **c** *S. luteoviridis*; **d** *S. wendelboi*; **e** *S. florulenta*; **f** *S. aizoides*. Note the difference in size between the guard cells of the stomata on the left and those of the hydathode in the center of **c**. Lime incrustations in **a**, **c** and **d** removed by treatment with citric acid. Scale bars represent 100 μm. *h* hydathode; *st* leaf area with stomata

The lime-secreting hydathodes in saxifrages first extrude the mineral probably as calcium hydrogen carbonate [Ca(HCO₃)₂]. As the water evaporates outside, the calcium carbonate (CaCO₃) crystallizes and forms a crust over the pore. This crust may possibly hinder the gutting process and water loss (Takeda et al. 1991). Sometimes, crystals fill the entire pit above the pore, and large carbonate crusts, which are flaky or sometimes funnel-shaped, rise above the leaf’s surface in the hydathode area (Figs. 1, 2a, b, 3b). It has also been speculated that stomata release calcium bicarbonate solution, albeit to a lesser extent than hydathodes do (Michavila et al. 2021). This was thought to explain why a calcium carbonate crust sometimes covers the entire leaf surface. However, the release of an aqueous solution through the stomata implies that the mesophyll intercellular spaces would have to be filled with water, which is unlikely as it would prevent photosynthesis. Therefore, earlier researcher’s observations (Unger 1861; Waldner 1878) that the crusts on the epidermis of the upper and lower leaf surfaces of *S. crustata*, *S. hostii* (as *S. elatior*) and *S. paniculata* (as *S. aizoon*) are produced in young leaves by the parenchyma before the hydathodes begin to guttate appear more likely. However, according to our observations, flat, white coatings consisting of thin plates first appear on the leaf surfaces around the hydathodes, particularly at the leaf tips. Then, they spread further (Fig. 1c, d, f, g). In some species, these coatings can develop into dense, flat, sometimes flaking, lime patches on the upper side of the leaf (Fig. 1a, f). Apparently, the mineral supply for leaves with heavy incrustation comes from the guttating hydathodes (Figs. 1a, g, 2a, b), whereby the guttation fluid spreads across the leaf surface.

The lime-secreting function of the hydathodes may represent an adaptive trait connected with the regulation of internal Ca^2^⁺ concentration. It may also affect light deflection properties of the leaf margin, which could aid in light capture by the mesophyll (Kerker effect) or protect against excessive insolation. In alpine or mountainous environments, this function can counteract evapotranspiration and drought- or wind-induced dehydration, limit the abrasive effects of snow and ice in winter, reduce freeze–thaw cycle-induced water loss, and serve as a defense mechanism against herbivores and parasites, as lime-encrusted plants experience significantly less infestation. Additionally, plants can secrete pollutants through guttation and maintain water flow when transpiration is absent (Braun 1913; Horný et al. 1986; Webb and Gornall 1989; Kaplan 1995; Neuner et al. 1999; Hacker and Neuner 2006; Wightman et al. 2018; Michavila et al. 2021).

In order to study the diversity of the spatial arrangement of stomata and lime-secreting hydathodes, as well as their cellular structure, in the leaves of *Saxifraga* in more detail, this study includes species from different phylogenetic groups using light and scanning electron microscopy (SEM). The study focuses on the species-rich section *Porphyrion*, divided into several subgroups comprising a total of approximately 102 species, of which 58 were examined. From the much less species-rich section *Ligulatae*, with a total of probably 14 species, 10 species were examined. The aim of this sampling was to record the structural diversity and spatial distribution of both organs within these phylogenetic groups, relating them to the species’ site, ecological, and geographical characteristics where appropriate.

This research approach initially required special attention to methodological problems arising from the analysis and representation of cellular structures, which were often heavily calcified. Additionally, preparing the plant samples, many of which were old herbarium specimens of rare species, was time-consuming.

## Material and methods

### Plant specimens and tissue pretreatment

A total of 85 plant samples (70 species, 7 subspecies or varieties, 2 hybrids and 6 multiple accessions) were examined and evaluated using light microscopy and scanning electron microscopy (SEM). The plant material used for morphological studies (Table 1; Suppl. Table S1) consisted of adult, fully developed, intact and undamaged leaves. Fresh leaf samples were collected in Germany and the Czech Republic between February and March 2023. Other samples were from the holdings of Botanical Gardens and private saxifrage collections. The dried leaf material of additional species came from specimens of various herbaria. Details on the collections of the analyzed species can be found in the Suppl. Table S1.

**Table 1:** Lime-secreting hydathodes and occurrence of amphistomatic or hypostomatic leaves in the studied *Saxifraga* species of the sections *Ligulatae* and *Porphyrion*, providing the number of hydathode pits per leaf, their location on the adaxial leaf surface and the stomatal arrangement on the leaf surfaces. The number of studied species/total species number in each section and subsection is given in brackets. *amphi* amphistomatic; *hypo* hypostomatic; *N/A* not available

Taxon	Number of pits per leaf	Location of pits on the adaxial leaf surface	Number of hydathodes per pit	Leaf type
sect. *Ligulatae* Haw. (10/14)				
* S. callosa* Sm.	> 25	entire margin	4	amphi
* S. cartilaginea* Willd.	> 25	entire margin	1	N/A
* S. cochlearis* Rchb.	> 25	entire margin	2	amphi
* S. cotyledon* L.	> 25	entire margin	3	amphi
* S. crustata* Vest	> 25	entire margin	2	hypo
* S. hostii* Tausch	> 25	entire margin	2	amphi
* S. kolenatiana* Regel	> 25	entire margin	1	N/A
* S. lingulata* Bellardi	> 25	entire margin	4	amphi
* S. paniculata* Mill.	> 25	entire margin	2	amphi
* S. valdensis* DC.	3–5	distal 2/3	NA	N/A
sect.* Porphyrion* Tausch (58/102)				
subsect. *Florulentae* (Engl. & Irmsch.) Gornall (1/1)				
* S. florulenta* Moretti	3–10	distal 2/3	1	amphi
subsect. *Kabschia* (Engl.) Rouy & Camus (51/93)				
* S. alberti* Regel & Schmalh.	3–5	distal 1/3	1	amphi
* S.*× *alpigena* Harry Sm. (= S. *andersonii* × *quadrifaria*; fide Bürgel 2007)	1	leaf tip	1	amphi
* S. andersonii* Engl.	5–7	distal 1/2	1	amphi
* S. aretioides* Lapeyr.	3–5	distal 1/2	1	amphi
* S. burseriana* L.	5–7	entire margin	1	amphi
* S. caspica* Sipliv.	3	distal 1/3	1	N/A
* S. caucasica* Sommier & Levier	1–3	distal 1/2	1	N/A
* S. charadzeae* Otsch.	5–7	distal 3/4	1	amphi
* S. cinerea* Harry Sm.	11–13	entire margin	1	hypo
* S. columnaris* Steud.	3–5	distal 1/3 to 1/2	1	amphi
* S. corymbosa* Boiss.	7–15	entire margin	1	N/A
* S. desoulavyi* Oett.	1–5	distal 1/3 to 1/2	1	amphi
* S. diapensioides* Bellardi	5–7	distal 1/2 to 2/3	2	amphi
* S. dinnikii* Schmalh. ex Akinf.	3–7	distal 1/3 to 1/2	1	hypo
* S. duthiei* Gand.	3	leaf tip	1	amphi
* S. federici*-*augusti* Biasol. subsp. *federici*-*augusti*	15–25	entire margin	1	amphi
* S. federici*-*augusti* subsp. *grisebachii* (Degen & Dörfl.) D.A.Webb	15– > 25	entire margin	1	amphi
* S. felineri* P.Vargas	7–9	distal 2/3	2	amphi
* S. ferdinandi*-*coburgi* Kellerer & Sünd.	5–7	entire margin	2	amphi
* S. georgei* J.Anthony	1	leaf tip	1	amphi
* S. iranica* Bornm.	3–5	distal 1/3 to 1/2	2	amphi
* S. juniperfolia* Adams	3–7	distal 1/2	1	amphi
* S. karadzicensis* (Degen & Košanin) Bürgel	1	leaf tip	1	amphi
* S. kotschyi* Boiss.	7–13	distal 2/3	2	amphi
* S. lilacina* Duthie	5	distal 1/2	1	hypo
* S. ludlowii* Harry Sm.	3–5	leaf tip	2	amphi
* S.* × *luteopurpurea* Lapeyr.	15–17	entire margin	1	amphi
* S. luteoviridis* Schott & Kotschy	7–17	entire margin	1	amphi
* S. marginata* Horný	3–13	distal 2/3	3	amphi
* S. marginata* var. *bubakii* Horný	7–13	distal 2/3	3	amphi
* S. matta*-*florida* Harry Sm.	1–2	leaf tip	1	amphi
* S. meeboldii* Engl. & Irmsch.	5–7	distal 1/3 to 1/2	1	amphi
* S. nana* Engl.	1(–3)	leaf tip	2	amphi
* S. ovczinnikovii* Kamelin	5	distal 1/3	1	amphi
* S. porophylla* Bertol.	5–11	entire margin	1	N/A
* S. pseudolaevis* Oett.	5–9	distal 1/2 to 2/3	1	amphi
* S. pulvinaria* Harry Sm.	1	leaf tip	1	amphi
* S. quadrifaria* Engl. & Irmsch.	1	leaf tip	1	amphi
* S. ramsarica* Jamzad	5	distal 1/2	1	amphi
* S. roylei* Harry Sm.	1	leaf tip	1	amphi
* S. ruprechtiana* Manden.	5–7	distal 1/2 to 2/3	1	N/A
* S. sancta* Griseb.	3–5	distal 1/2	1	amphi
* S. scardica* Griseb.	7–15	distal 3/4	1	amphi
* S. scleropoda* Sommier & Levier	1–5	distal 1/3 to 1/2	1	amphi
* S. sempervivum* C.Koch	9–21	entire margin	1	amphi
* S. spruneri* Boiss.	3–5	distal 1/3	1	amphi
* S. spuneri* var. *deorum* (Pénzes) Horný & Webr	3	distal 1/2	1	amphi
* S. stolitzkae* Duthie ex Engl. & Irmsch.	7–13	distal 1/2	1	amphi
* S. stribrnyi* (Velen.) Podp.	17–25	entire margin	2	amphi
* S. subverticillata* Boiss.	5–7	distal 1/3 to 1/2	1	amphi
* S. thessalica* Schott, Nym. & Kotschy	11	entire margin	1	amphi
* S. tombeanensis* Boiss. ex Engl.	1–3	leaf tip	1	amphi
* S. unguipetala* Engl. & Irmsch.	5–7	distal 1/2	1	amphi
* S. unifoveolata* Slipliv.	1	leaf tip	1	amphi
* S. vandellii* Sternb.	5–7	distal 1/2	2	amphi
* S. wendelboi* Schönb.-Tem.	9–13	distal 2/3	3	amphi
subsect. *Mutatae* (Engl. & Irmsch.) Gornall (2/2)				
* S. aizoides* L.	1–5	distal 1/3 to 1/2	5–7	amphi
* S. mutata* L.	> 25	entire margin	1	amphi
subsect. *Oppositifoliae* Hayek (3/4)				
* S. biflora* All.	1–3	leaf tip	2	amphi
* S. oppositifolia* L.	1–3	leaf tip	1	amphi
* S. oppositifolia* subsp. *oppositifolia*	1–3	leaf tip	1	amphi
* S. oppositifolia* subsp. *asiatica* (Hayek) Engl. & Irmsch.	1–3	leaf tip	1	amphi
* S. oppositifolia* subsp. *smalliana* (Engl. & Irmsch.) Hultén	1–3	leaf tip	1	amphi
* S. retusa* subsp. *augustana* (Vacc.) P.Fourn.	3–5	distal 1/2	1	hypo
subsect. *Squarrosae* (Engl. & Irmsch.) Tkach, Röser & M.H.Hoffm. (1/2)				
* S. caesia* L.	3–7	distal 1/3	1	amphi

To soften the leaves and restore their original spatial structure, the dried leaves of herbarium specimens were boiled in water in a microwave at 500 W for 90 s. This procedure was not carried out on fresh samples. After testing mechanical and thermal treatments, as well as several different acids (acetic and hydrochloric at various concentrations) to remove the lime crusts, the dry and fresh leaf samples were transferred to a 1 M citric acid solution for 5 min at room temperature. This treatment was the most effective because it dissolved the carbonate crusts on the leaf surfaces and in the hydathode pits (cavities) found in many species. After the lime had dissolved in the acid, the samples were transferred to distilled water to remove any residual acid. At least two and at most three leaf samples were prepared for each species. To document the lime crusts and for comparison purposes, one leaf sample per species was not treated with the citric acid solution. The herbarium samples were transferred directly to distilled water after boiling, while the fresh samples were transferred immediately.

### Preparation for scanning electron microscopy (SEM)

After removing the incrustations, the samples were dehydrated using an ascending series of alcohols: 30%, 50%, 70%, 90%, and 96%. The samples were placed in ethanol solutions at each concentration for 10 min. The samples were then transferred to the pressure chamber of a critical point dryer (CPD 030; BAL-TEC, Balzers Union, Balzers, Liechtenstein). Initially, the chamber was filled with 96% undenatured ethanol, which was replaced with CO_2_ that was finally released from the chamber. The samples were dry but hygroscopic and were stored in an air-dry environment (e.g., with silica gel) or processed further with the sputter coater.

Dry samples suitable for electron microscopy were mounted on aluminum stubs using double-stick carbon conductive tabs (Plano GmbH, Wetzlar, Germany) and coated with gold in an argon atmosphere using a sputter coater (MED 010, Balzers Union). Alternatively, samples were studied as wet samples under low vacuum at −25 °C using coolstage equipment (Deben UK Ltd., London, UK).

The samples were transferred to a Hitachi TM-3030Plus tabletop SEM (Hitachi Europe Ltd., Maidenhead, UK) and scanned at an acceleration voltage of 5 kV. Images were taken at magnifications ranging from 50 to 2500 times.

## Results

### Arrangements of stomata on the leaves

**Fig. 4:**
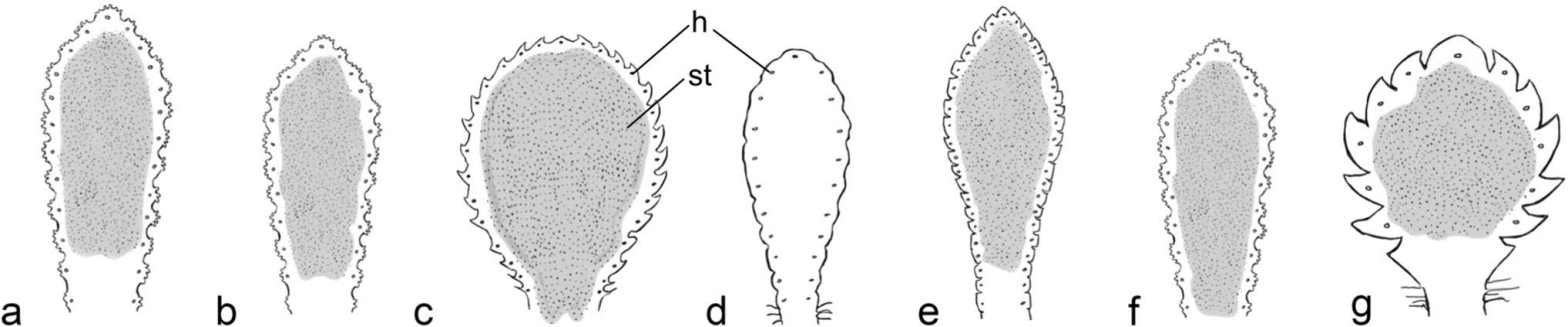
Arrangement of stomata and location of lime-secreting hydathode pits on the adaxial surface of the basal leaves of *Saxifraga* sect. *Ligulatae* species in schematic representation. **a** *S. callosa*; **b** *S. cochlearis*; **c** *S. cotyledon*; **d** *S. crustata*; **e** *S. hostii*; **f** *S. lingulata*; **g** *S. paniculata*. Leaves not drawn to scale. *h* hydathode; *st* leaf area with stomata (shaded)

**Fig. 5:**
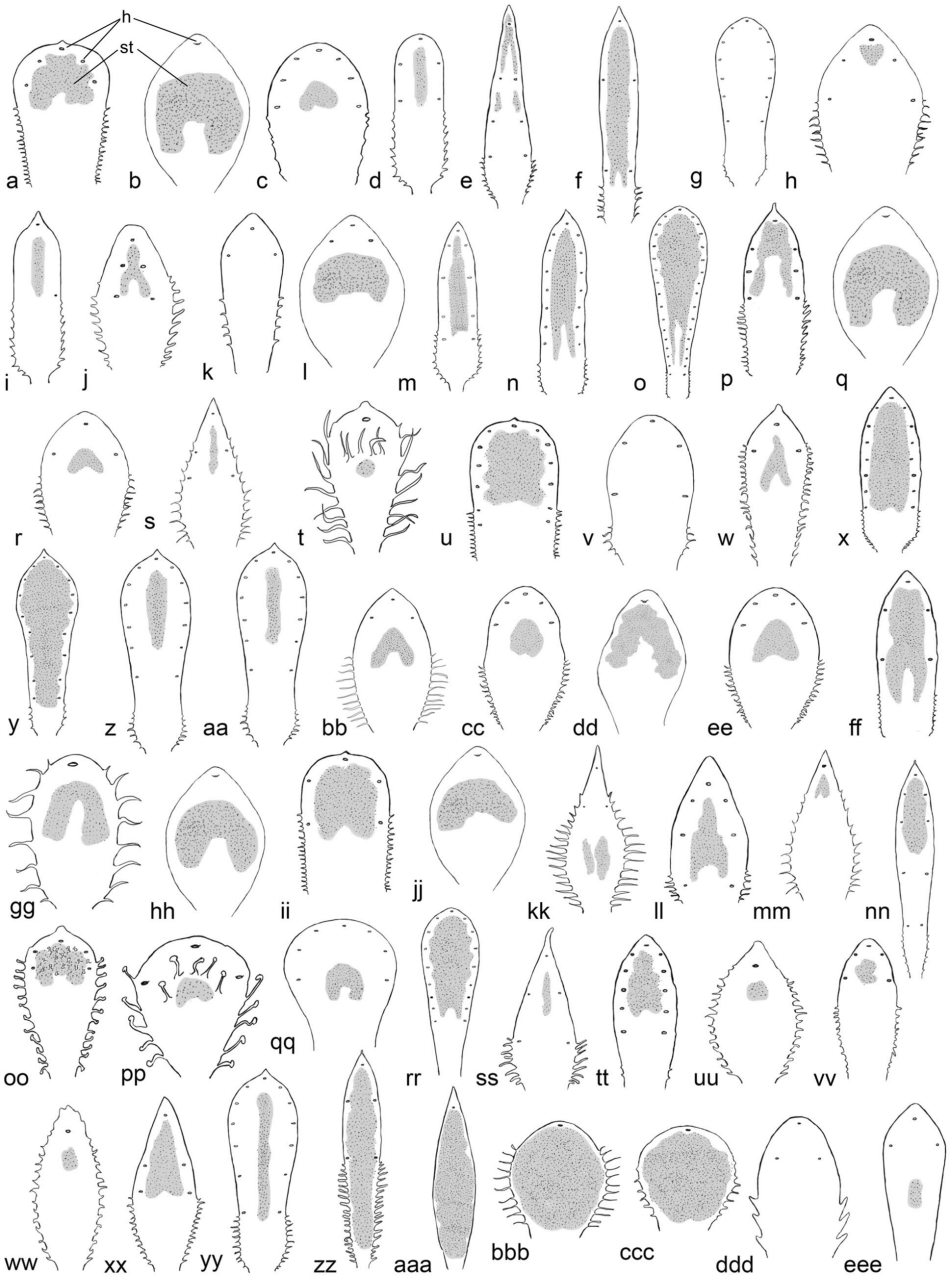
Schematic drawing of the arrangement of stomata and location of lime-secreting hydathode pits on the adaxial surface of the basal leaves of *Saxifraga* sect. *Porphyrion* subsect. *Kabschia* (**a–yy**), subsect. *Florulentae* (**zz**), subsect. *Mutatae* (**aaa**), subsect. *Oppositifoliae* (**bbb–ddd**) and subsect. *Squarrosae* (**eee**). **a** *S. alberti*; **b** *S. alpigena*; **c** *S. andersonii*; **d** *S. aretioides*; **e** *S. burseriana*; **f** *S. charadzeae*; **g** *S. cinerea*; **h** *S. columnaris*; **i** *S. desoulavyi*; **j** *S. diapensioides*; **k** *S. dinnikii*; **l** *S duthiei*; **m** *S. felineri*; **n** *S. federici-augusti* subsp. *federici-augusti*; **o** *S. federici-augusti* subsp. *grisebachii*; **p** *S. ferdinandi-coburgi*; **q** *S. georgii*; **r** *S. iranica*; **s** *S. juniperifolia*; **t** *S. karadzicensis*; **u** *S. kotschyi*; **v** *S. lilacina*; **w** *S. ludlowii*; **x** *S.* × *luteopurpurea*; **y** *S. luteoviridis*; **z** *S. marginata;* **aa** *S. marginata* var. *bubakii*; **bb** *S. matta-florida;* **cc** *S. meeboldii*; **dd** *S. nana*; **ee** *S. ovczinnikovii*; **ff** *S. pseudolaevis*; **gg** *S. pulvinaria*; **hh** *S. quadrifaria*; **ii** *S. ramsarica*; **jj** *S. roylei*; **kk** *S. sancta*; **ll** *S. scardica*; **mm** *S. scleropoda*; **nn** *S. sempervivum*; **oo** *S. spruneri*; **pp** *S. spruneri* var. *deorum*; **qq** *S. stolitzkae*; **rr** *S. stribrnyi*; **ss** *S. subverticillata*; **tt** *S thessalica*; **uu** *S. tombeanensis*; **vv** *S. unguipetala*; **ww** *S. unifoveolata*; **xx** *S. vandellii*; **yy** *S. wendelboi*; **zz** *S. florulenta*; **aaa** *S. aizoides*; **bbb** *S. biflora*; **ccc** *S. oppositifolia* subsp. *asiatica*; **ddd** *S. retusa* subsp. *augustana*; **eee** *S. caesia*. Leaves not drawn to scale. *h* hydathode; *st* leaf area with stomata (shaded)

**Fig. 6:**
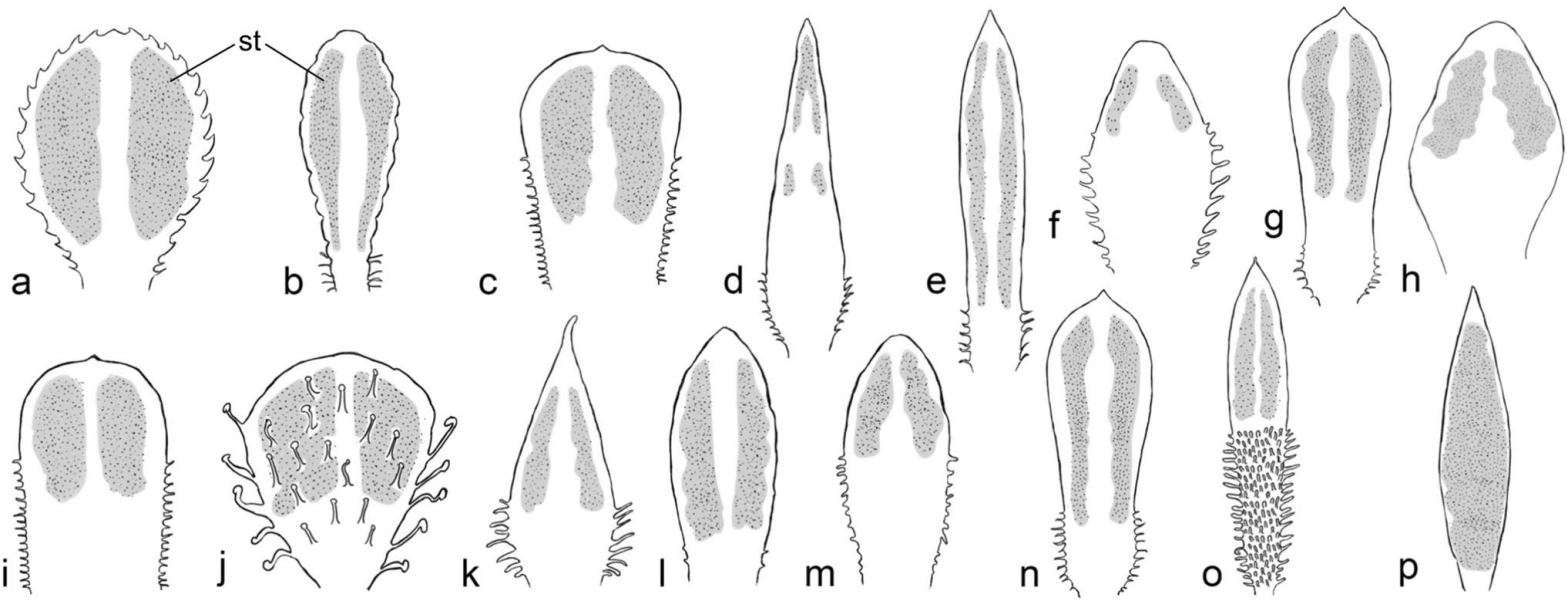
Schematic representation of the arrangement of stomata on the abaxial surface of the basal leaves of *Saxifraga* sect. *Ligulatae* (**a–b**), sect. *Porphyrion* subsect. *Kabschia* (**c–n**), subsect. *Florulentae* (**o**) and subsect. *Mutatae* (**p**). **a** *S. cotyledon*; **b** *S. crustata*; **c** *S. alberti*; **d** *S. burseriana*; **e** *S. charadzeae*; **f** *S. diapensioides*; **g** *S. marginata*; **h** *S. nana*; **i** *S. ramsarica*; **j** *S. spruneri* var. *deorum*; **k** *S. subverticillata*; **l** *S. thessalica*; **m** *S. unguipetala*; **n** *S. wendelboi*; **o** *S. florulenta*; **p** *S. aizoides*. Leaves not drawn to scale. *h* hydathode; *st* leaf area with stomata (shaded)

All 66 species and subspecies of *Saxifraga* studied using SEM consistently had anomocytic stomata. This means that no subsidiary cells with a particular shape, arrangement, or number that differ from other epidermal cells were recognizable. 61 of them were amphistomatic, and only 5 were hypostomatic (Table 1; Figs. 4, 5 and 6). They lacked stomata on the adaxial leaf surface and only had them abaxially. Some recurrent patterns and correlations between leaf shape and the number of hydathode pits were identified among species with amphistomatic stomata. The size of the individual leaf played a minor role and did not impact the basic pattern of hydathode number or their arrangement.

### Amphistomatic leaves

#### Adaxial (upper) leaf surface

- (1) Stomata mostly occurred along the entire adaxial leaf surface in the species with long spatulate leaves, crenate or dentate (toothed) leaf margins, and usually more than 25 hydathode pits were located along the entire margin or at the base of the teeth at the margin (Figs. 1a, 2a, b, 4). These species taxonomically belonged to the section *Ligulatae*. Exceptions included *S. crustata* and *S. valdensis*, as the former was hypostomatic (Figs. 4d, 6b) and lacked stomata on the upper leaf surface (see below) and the latter only had 3–5 pits per leaf (Table 1). A mostly circular zone around the pits was devoid of stomata. The approximate radius of these zones was the distance from the pit to the leaf margin (Fig. 4). This stomata-free zone may be related to the hydathode structure and its epithem cells at the ends of the xylem strands. There is no photosynthetic parenchyma tissue in this area that would require stomata for gas exchange. However, this phenomenon can only be observed on the adaxial side with the palisade parenchyma, as there are no corresponding circular stomata-free areas on the underside of the leaf with spongy mesophyll.
- (2) The stomata typically occurred only on the distal half or distal two-thirds of the adaxial leaf surface in the species, which had only one hydathode pit close to the tip of the leaf, such as *S. nana* (Table 1; Figs. 1e, 2f, 5dd). The stomata were arranged in the shape of an inverted horseshoe, and the area they occupied could vary in size and approximate a semicircle. The area occupied by the stomata was relatively large compared to the mentioned species with more pits. This could be due to the influence of the pits on the occurrence of stomata as seen in *S. alpigena*, *S. georgii*, *S. nana*, *S. quadrifaria* and *S. roylei* (Fig. 5b, q, dd, hh, jj). In *S. aizoides* specimens with hydathodes only at the tips of its lanceolate leaves, nearly the entire adaxial leaf surface was covered with stomata (Figs. 5aaa, 6p). However, this was not the norm. For example, *S. tombeanensis* and *S. unifoveolata* also had only one apical hydathode pit, but only a comparatively small area was covered with stomata. This could be linked to the teeth found on the margins of their leaves (Fig. 5uu, ww). However, *S. biflora* and *S. oppositifolia* subsp. *asiatica* share the occurrence of only one apical pit, yet their stomata were distributed across the entire leaf surface (Table 1; Figs. 1h, 5bbb, ccc). Therefore, there is no obvious reason for the highly variable stomatal distribution of leaves with only one apical pit.
- (3) In species with three to five hydathode pits on the distal half or two-thirds of the leaf (Figs. 1b–d, 5c, r, z, aa) the shape of the adaxial leaf surface covered by stomata was usually triangular with rounded tips, or resembled an arrowhead. Stomata primarily occurred on the distal half of the adaxial leaf surface, mostly distally to the proximal pits (Fig. 5c, d). Occasionally, however, they extended farther down toward the base of the leaf. Interestingly, species with teeth or trichomes at the leaf margin had stomata-covered areas only distal to the leaf region with these appendages (Fig. 5a, f, i, j, xx). It is unclear why this occurs, especially since these structures are found only at the leaf margin and do not affect the parenchyma tissue. This is all the more striking given that the formation of stomata in the vicinity of hairs on the leaf surface is unaffected in species with such hairs, as exemplified by *S. karadzicensis* and *S. spruneri* (Figs. 2e, 5t, oo, pp).
- (4) In species with lanceolate or spatulate leaves, and seven to more than 25 hydathode pits, but with an entire margin instead of a crenate or toothed one, the pits were located close to the leaf margin on the adaxial leaf surface. This arrangement contrasts with that describe above (1), and the species in question taxonomically belonged to sect. *Porphyrion*. The stomata arrangement on the adaxial side of the leaf extended from the tip to the base, covering most of the leaf surface and following the pattern described above (1) for the sect. *Ligulatae* species. Examples included the two subspecies of *S. federici-augusti*, *S.* × *luteopurpurea*, *S. luteoviridis*, *S. stribrnyi* and *S. wendelboi* (Fig. 5n, o, x, y, rr, yy).

#### Abaxial (lower) leaf surface

To allow comparison, SEM examinations were carried out on 16 example species to study the location and distribution of stomata on the undersides of leaves (Fig. 6). Of these species, all but one were amphistomatic; only *S. crustata* was hypostomatic (Table 1; Figs. 4d, 6).

The distribution of stomata on the abaxial side of the leaf showed a largely recurrent pattern in all species. Stomata occurred symmetrically on both longitudinal halves of the leaf lamina. No stomata occurred abaxially above the midrib of the leaf blade, as usual in leaves. The lengthwise extension of areas covered with stomata on the abaxial leaf surface resembled that of the adaxial side. If the stomata were located proximal to the most distal hydathode pit on the adaxial side, then there would also be stomata proximal to it on the abaxial side of the leaf, as seen, e.g., in *S. nana* (Figs. 5dd, 6h).

The most distal stomata on the upper and lower surfaces of the leaf were always in a certain distance from the tip. On the abaxial side, the stomata were arranged along the leaf margins toward the base but usually ended at the same height as those on the adaxial surface. If the leaf margin had teeth or trichomes, the stomata were located a certain distance from them, as they were on the adaxial surface (Figs. 5, 6).

#### Deviating patterns

However, there were several exceptions to the typical distribution of stomata on the upper and lower leaf surfaces:

- (1) On the adaxial side of *S. andersonii* leaves, stomata occurred only in a small area near the center of the leaf (Fig. 5c). On the underside, however, the stomata were distributed on both sides of the midrib over a large area covering the distal two-thirds of the leaf lamina (not shown).
- (2) In *S. sancta*, the stomata were located in the center of the adaxial leaf surface, which was far below the proximal pair of hydathode pits (Fig. 5kk). Additionally, the distribution of the stomata did not appear to be influenced by the trichomes of the leaf margin.
- (3) The stomata of *S. burseriana* were not located in the center of the adaxial leaf surface, but rather between the pits along the leaf margin (Figs. 2g, 5e). Even after being interrupted by a pair of pits, the stomata-covered regions continued along the leaf margin. *Saxifraga burseriana* was the only species with interrupted stomata regions on both the upper and lower leaf surfaces. Additionally, the stomata were distributed almost identically on both sides of the leaf (Figs. 5e, 6d).

### Hypostomatic leaves

Among species with long, spatulate leaves and more than 25 hydathode pits at the leaf margin, the section *Ligulatae* (see above), *S. crustata* was the only species that lacked stomata on its entire adaxial leaf surface (Fig. 4d). It was hypostomatic, and the stomata were present abaxially in two stripes close to the leaf margin along most of the leaf’s length (Fig. 6b).

The other hypostomatic species found in this study, i.e., *S. cinerea*, *S. dinnikii*, *S. lilacina* and *S. retusa* subsp. *augustana* (Figs. 5g, k, v, ddd) belonged to section *Porphyrion.* Most other studied species in this section had amphistomatic leaves (see above). The exclusive occurrence of stomata on the underside of the leaves in the four species did not appear to be related to the shape of their leaves or the number of their hydathode pits on the adaxial side of the leaves. This was evident from comparison with species having similarly shaped leaves. For example, amphistomatic *S. diapensioides* (Figs. 5j, 6f) and *S. duthiei* (Fig. 5l) had the same leaf outline as hypostomatic *S. dinnikii* (Fig. 5k) and *S. lilacina* (Fig. 5v), respectively. However, *S. diapensioides* and *S. lilacina* typically had five hydathode pits on the adaxial surface, whereas *S. dinnikii* and *S. duthiei* had only three. On the other hand, *S. aretioides* (Fig. 5d) and *S. stolitzkae* (Fig. 5qq) had five or more pits, similar to hypostomatic *S. lilacina* and *S. cinerea* (Fig. 5g), but they were amphistomatic. Furthermore, the leaf shape of the hypostomatic *S. retusa* subsp. *augustana* (Fig. 5ddd) also resembled that of the closely related *S. biflora* (Fig. 5bbb) and *S. oppositifolia* subsp. *asiatica* (Fig. 5ccc), which were amphistomatic. Therefore, the shape and outline of the leaf as well as the number of hydathode pits do not appear to be causally connected to amphi- or hypostomaty of the leaves.

### Hydathodes

The hydathodes of all studied species were similar in that they had kidney-shaped guard cells that resembled stomatal guard cells, but were larger (Fig. 3c). The hydathodes were typically sunk in deep pits (Fig. 3a–d), except for *S. aizoides* (Fig. 3f), *S. florulenta* (Fig. 3e) and *S. mutata* (Fig. 1g). In *S. aizoides*, the most distal hydathodes of the leaves were located close to the mostly apiculate leaf apex and nearly flush with the surface (Figs. 3f, 5aaa). In the latter two species, the hydathodes were in small, comparatively shallow pits close to the leaf margin. The leaf blade is very thin in these species at these points, so deep pits cannot form (Figs. 1g, 3e, 5zz).

The shape, width and depth of the pits with hydathodes were mostly characteristic of each species. The greatest variation among species was found in the sect. *Porphyrion*. Apart from *S. aizoides*, *S. florulenta* and *S. mutata*, the pits’ shapes ranged from extensive depressions (*S. nana*) to narrow, deep pits (*S. sancta*). The species from sect. *Ligulatae* were characterized by comparably large, cylindrical pits with mostly flat bottoms (Fig. 3a). This characteristic was also found in some species with elongate, obtuse, or spatulate leaves and a comparatively high number of pits, which belong to the sect. *Porphyrion*, such as *S. kotschyi*, *S. stolitzkae* and *S. wendelboi* (Fig. 3d).

The number of hydathodes per pit varied. Most species had only one hydathode per pit (Table 1; Fig. 3c, e, f). However, several species had 2–4 hydathodes per pit (Table 1; Fig. 3a, d). In *S. aizoides*, there were even up to seven hydathodes that could be found in one place. They were located in pits or, in the apical ones, were flush with the leaf surface distal to the mostly apiculate leaf tip. Five hydathodes could be arranged like the dots on a dice (Fig. 3f). Most section *Porphyrion* species had only one hydathode per pit, such as *S. florulenta* (subsect. *Florulentae*) and *S. squarrosa* (subsect. *Squarrosae*). Subsect. *Kabschia* species had 1–3 and subsect. *Mutatae* had 1–5 hydathodes per pit. All seven species of the sect. *Ligulatae* studied had more than one. Most had two to four hydathodes situated on the flat bottom of the usually large cylindrical pits (Table 1). At least three, and often four, hydathodes were present in the numerous pits on the leaf margin of *S. callosa*, *S. cotyledon* and *S. lingulata* (Fig. 3a).

## Discussion

The genus *Saxifraga* has always attracted the attention of botanists because some of its approximately 480 species have conspicuous, variable-numbered, dot-shaped, or extensive white, calcareous patches on their leaves. One of the respective species groups, the section *Ligulatae*, is popularly known as the “Silver saxifrages” in English. These characteristics are caused by hydathodes, which are traditionally referred to as lime-secreting or chalk glands (Metcalfe and Chalk 1950). The hydathodes probably secrete calcium bicarbonate, which is dissolved in the xylem sap and converts to calcium carbonate when the water evaporates (Figs. 1, 2a, b, 3b, e).

The hydathodes are epithem hydathodes, which also occur in other saxifrages, but in lime-secreting species they have the special feature that their guard cells are sunk to the bottom of a pit extending from the leaf surface into the leaf parenchyma. Calcium crystals first form in this pit (Fig. 3b) and then, depending on the location of the hydathodes, become visible in increasing quantities on the leaf surface or at the leaf margin as conspicuous, raised, white crystal accumulations (Figs. 1). In some species, these accumulations cover the entire leaf (Figs. 1a, 2a, b). The reason why these lime-secreting hydathodes, unlike the water-secreting ones, have these pits is unknown.

The subject of our investigation was to study the occurrence of hydathodes on leaves, their extraordinary variation in number and arrangement, a possible connection with the location of stomata, and their evolutionary origin, referring to the latest findings on the phylogeny of saxifrages. To this end, we carried out light and scanning electron microscopic examinations (SEM) of the leaf surfaces of fresh and dried leaf material (herbarium vouchers), which included an initial pretreatment to prepare it for examination, particularly to remove the calcareous incrustations (see Material and methods).

In connection with SEM investigations of leaf surfaces as well as pit and hydathode distributions, new findings emerged: there is highly variable stomatal distribution on the adaxial and abaxial sides of leaves among species.

### Stomata

All of the studied *Saxifraga* samples had anomocytic stomata, thus confirming previous studies (Moreau 1971, 1984; Webb and Gornall 1989). These stomata, also called the Ranunculaceae type, are furthermore widespread in other eudicots, including Aceraceae, Asteraceae, Berberidaceae, Cucurbitaceae, Plantaginaceae, Primulaceae and Scrophulariaceae (Van Cotthem 1970; Jurzitza 1987; Leistner and Breckle 2014; Dingermann et al. 2016). The kidney-shaped guard cells, with their wall thickenings and obliquely inward movement (Fig. 3), agree with the *Helleborus* type. This type occurs not only in saxifrages but also in other eudicots (Jurzitza 1987).

In most of the sampled species, the stomata were arranged on the adaxial and abaxial surfaces (Table 1; Figs. 2c–h, 4–6). This amphistomatic distribution is typical of species adapted to high light intensities, which fits the habitats of most of the species, which are often found in rocky areas in mountainous regions, frequently above the timberline. The areas around the hydathode pits always lack stomata, presumably because there are no photosynthetically active mesophyll cells in the region of the xylem endings beyond the hydathodes, making gas exchange unnecessary. The arrangement of stomata seems to be partially caused by the presence of one apical hydathode, three to five hydathodes, or more than 25 hydathodes along the leaf margin, usually at the base of the leaf’s teeth or tooth-like appendages (Figs. 2a, b, 4, 6a, b). The precise shape of regions with stomata mostly follows some recurrent patterns but no general rules, and it is species-specific in most cases. However, presumably related species showed similar patterns.

The stomatal distribution on the underside of the leaf (Fig. 6) was generally similar to that on the upper surface as can be seen in *S. burseriana* (Figs. 2g, 5e, 6d) and *S. diapensioides* (Figs. 2h, 5j, 6f) by comparing both leaf surfaces*.* However, differences in detail were apparent in some species (Figs. 4, 5). Similar to the adaxial surface, the distribution of photosynthetically active mesophyll cells in the interior of the leaf appears to be the basis for the congruent stomatal distribution on the abaxial leaf surface.

In rare cases, the leaves of the studied *Saxifraga* species were hypostomatic rather than amphistomatic. This was observed in a few species from both major taxonomic groups, the sections *Ligulatae* and *Porphyrion* (Figs. 4d, 5g, v, ddd, 6b).

### Hydathodes

The species of sections *Ligulatae* and *Porphyrion* differed in terms of the diameter and shape of the hydathode pits. While most species had only one hydathode per pit was presents, occasionally, up to five hydathodes could be found (Table 1, Figs. 4, 5). This appeared to correlate with pit diameter. A higher number of hydathodes was present in the large cylindrical pits of *Ligulatae* species (Fig. 3a), as was observed previously for *S. cochlearis* in this section (Wightman et al. 2017). However, up to seven hydathodes were found in the at most shallow yet extensive deepenings of *S. aizoides* from subsect. *Mutatae* (Fig. 3f).

### Ecological and phylogenetic context

#### Ecology

The found presence of hypostomatic leaves in otherwise consistently amphistomatic taxonomic groupings, namely *S. crustata* in sect. *Ligulatae* and *S. cinerea*, *S. dinnikii*, *S. retusa* subsp. *augustana* and *S. lilacina* in sect. *Porphyrion*, cannot be explained by ecological or environmental factors. *Saxifraga crustata* is found on limestone and dolomitic screes and rocks between the montane and the alpine zones from the eastern Alps to the mountains of the northwestern Balkan Peninsula and does not differ significantly from other members of sect. *Ligulatae*, which mostly occur in similar habitats and mountainous regions (Webb and Gornall 1989). However, these species are amphistomatic.

The hypostomatic species *S. cinerea*, *S dinnikii* and *S. lilacina* are rock dwellers in the subalpine to alpine zones. They are distributed from the Caucasus to the Himalayas and are apparently not much different from the presumably closely related, amphistomatic species in subsection *Kabschia* (Horný et al. 1986).

A similar observation applies to the hypostomatic species *S. retusa* subsp. *augustana*, which grows at high elevations on calcareous and base-rich substrates. Its habitat is comparable to that of its close relatives in subsect. *Oppositifoliae*, especially *S. oppositifolia* (Webb and Gornall 1989), which is amphistomatic. At least, this is true for the studied subsp. *asiatica*. As previously noted for several plant genera from different families (Wagner 1892), the frequent occurrence of amphistomatic leaf architecture in high-altitude habitats with strong insolation seems to be confirmed by our sampled saxifrages. However, there were exceptions that prevent generalization. In both primary taxonomic groups studied (sections *Ligulatae* and *Porphyrion*), hypostomatic leaves occurred in species whose leaves did not differ much from those of their amphistomatic relatives (see above).

Amphistomatic leaf architecture could be a way to overcome CO₂ limitations in photosynthesis (Mott et al. 1982; Drake et al. 2019). This architecture is also a prerequisite for photosynthetic activity on both sides of the leaf (see Introduction). This architecture may be linked to the internal anatomy of the leaf mesophyll and its photosynthetic capability, which is an interesting topic for future studies.

Additionally, many saxifrages have fleshy, thick, and rather succulent leaves, which may also be related to the demands of photosynthesis. However, this potential correlation has not yet been studied. The water uptake of saxifrages with amphistomatic and fleshy leaves may be limited to nighttime for species that are exposed to sunlight during the day and become heated and dried out. This could explain the increased guttation observed in *S. paniculata* leaves during the second half of the night (Schmidt 1930; as *S. aizoon*).

#### Phylogeny

Lime-secreting hydathodes in *Saxifraga* are taxonomically limited to the sections *Ligulatae* and *Porphyrion*. In some species, the calcareous crusts may be intense and cover parts or all of the leaf surface (Figs. 1a, 2a, b). Examples include *S. callosa*, *S. cochlearis*, *S. paniculata*, *S. longifolia* and *S. valdensis* in sect. *Ligulatae* as well as *S. caesia*, *S. diapensioides*, *S. ferdinandi-coburgi*, *S. squarrosa* and *S. stribrnyi* in sect. *Porphyrion*. The leaves of *S. valdensis* (sect. *Ligulatae*), especially, are heavily lime-encrusted. However, reports that this is due to hydathodes being present not only along the leaf margin but also scattered across the adaxial leaf surface are incorrect (Webb and Gornall 1989; McGregor 2008). All hydathodes are marginal or close to the margin.

In most cases, the incrustations are confined to the leaf margin or the adaxial surface of the leaf near the margin. In both cases, white, raised spots of crystals form. This is due to the activity of the epithem hydathodes, which are widespread in the genus *Saxifraga* (Zhang et al. 2015). However, only the hydathodes of the sections *Ligulatae* and *Porphyrion* produce these carbonate incrustations. These hydathodes are typically sunk into pits that extend deep into the leaf mesophyll. Unlike the hydathodes of other *Saxifraga* species, which secrete water and are flush with the surface, the hydathodes in these pits release dissolved calcium bicarbonate. The reason for this is not fully understood. Recent studies suggest that this is likely due to a selective mechanism of the epithem cells located at the ends of the xylem strands. This mechanism causes the hydrogen carbonate to be released preferentially from the xylem sap while retaining other dissolved substances (Cerutti et al. 2019; Bellenot et al. 2022; Fehlauer et al. 2022b).

In this context, it is interesting to note that, according to current molecular phylogenetic data, *S. florulenta* belongs to sect. *Porphyrion* rather than to sect. *Ligulatae*. It was traditionally classified under the latter due to its long, lanceolate leaves. However, it only switched its position between two typically lime-secreting sections. In our study, *S. florulenta* notably had no macroscopically recognizable lime incrustations on its leaves, which confirmed previous observations (McGregor 1980; Webb and Gornall 1989). However, scanning electron microscopy (SEM) revealed the presence of incrustations surrounding the hydathodes (Fig. 3e). *Saxifraga florulenta* is endemic to the Mercantour and Argentera regions of the Maritime Alps. It grows as a chasmophyte above 2,000 m altitude on siliceous gneiss and granite cliffs that are nearly devoid of vegetation and exposed to the north or northeast (McGregor 2008; Guerrina et al. 2020). Therefore, the absence of leaf incrustations in *S. florulenta* may also be related to the substrate’s low calcium content. In this respect, *S. florulenta* resembles the silicophilous *S. cotyledon* of the sect. *Ligulatae*, which also exhibits slight lime secretion, albeit stronger than *S. florulenta* (see Introduction above). The same applies to the similarly silicophilous *S. kolenatiana* in the Caucasus (N.T., personal observation), confirming McGregor (2008). Unfortunately, there are no results from cultivated specimens of *S. florulenta*, that would show whether the lime secretion of the hydathodes is also low when it was cultivated on a calcareous substrate. It is difficult to cultivate (Webb and Gornall 1989; McGregor 2008) and does not tolerate calcareous substrates in any form (Köhlein 1980). Therefore, the proposed cultivation on calcium-rich substrates is likely to fail anyway. Interestingly, *S. florulenta* exhibits the camptodromous leaf venation pattern characteristic of all other lime-secreting saxifrages (see below).

*Saxifraga aizoides* had groups of hydathodes in at most shallow deepenings at the tip of its leaves (Fig. 3f) as well as a variable number of up to four hydathode pits along the distal half of the leaf margin. Lime secretion in this species varied greatly, ranging from a complete absence of deposits to dot-like incrustations or even extended, patchy ones, as previously recorded (Webb and Gornall 1989). This species grows on various types of bedrock, but it avoids extremely base-poor sites. The production of occasionally very conspicuous calcium deposits is most likely related to substrates with high calcium content (M.R. and N.T., personal observations).

*Saxifraga mutata*, characterized by having more than 25 small, shallow pits, each with one hydathode, close to the leaf margin (Table 1; Fig. 1g). It is intimately related to *S. aizoides*, and the two species form the subsection *Mutatae*. *Saxifraga mutata* had sparse to conspicuous lime incrustations as previously noted (Kurt 1930), comparable to those of *S. aizoides*. It also occurs on a variety of substrates, a characteristic that seems to be responsible for the degree of lime secretion in *S. mutata* as it is in *S. aizoides*. In the species-rich subsection *Kabschia*, a further lineage within sect. *Porphyrion*, there are additional species that exhibit little to no lime secretion. These species include the aforementioned silicophilous *S. juniperifolia*, *S. lilacina*, and *S. pulvinaria* (see Introduction).

Most species of subsect. *Kabschia* are calciphilicous with respect to the bedrock and typically exhibit lime-secreting hydathode activity. However, some calciphilous species have the typical pits with sunken hydathodes characteristic of lime-secreting species, but have only weak or absent lime incrustations. Examples include *S. burseriana*, which is found in the eastern Northern and Southern Limestone Alps, and *S. tombeanensis* and *S. vandellii*, which are found only in the central Southern Limestone Alps and grow in rock crevices (Webb and Gornall 1989). One possible explanation for their lack of lime secretion is that these species have very limited hydathode activity, resulting in minimal to no guttation due to limited water availability at their specific sites.

**Fig. 7:**
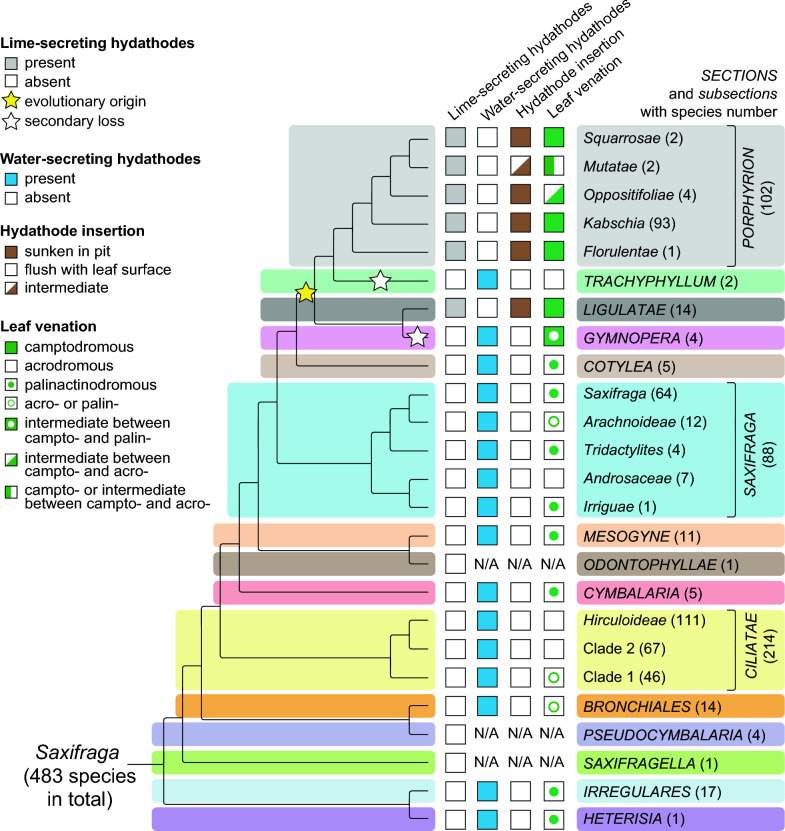
Cladogram depicting the phylogeny of the sections and subsections of the genus *Saxifraga* as well as their significant leaf characteristics. The columns provide information on hydathode types and locations, leaf venation and the approximate number of extant species included in the infrageneric taxa. The yellow star indicates the probable phylogenetic origin of the lime secretion syndrome, and the empty star indicates the loss of this ability (evolutionary reversal). Cladogram based on phylogenetic analyses of nuclear and plastid DNA sequences, as well as nuclear low-copy DNA loci (modified from Tkach et al. 2025). Leaf vascularization patterns according to Zhang et al. (2015) and our own results. *N/A* not available

Since lime-secreting hydathodes are found in only two of the 15 *Saxifraga* sections, one might ask whether this trait evolved once (Hypothesis 1) or twice (Hypothesis 2). In principle, a phylogenetic tree of *Saxifraga* encompassing all phylogenetic groupings (sections and subsections) could help answer this question (Fig. 7). According to Hypothesis 1, lime-secreting hydathodes must have originated in the last common ancestor (LCA) of the sections *Gymnopera*, *Ligulatae*, *Trachyphyllum* and *Porphyrion*. A similar suggestion has been made previously (Conti et al. 1996), however, section *Mesogyne* was included in this group, which is not supported by the current phylogenetic data (Fig. 7). It was argued by the authors that the LCA was calcicolous, which still remains plausible. According to Hypothesis 1, the lime secretion ability was lost twice: in the sections *Gymnopera* and *Trachyphyllum* (Fig. 7).

According to Hypothesis 2, lime-secreting hydathodes originated in the ancestor of sect. *Ligulatae* and in the ancestor of sect. *Porphyrion*. Support for this hypothesis could be that these hydathodes in these sections are located differently on the leaves. In sect. *Ligulatae*, they are mostly located at the base of sharp or rounded, convex teeth on the leaf margin, if the leaf margin is dentate or crenate (Figs. 1a, 2a, b, 4a–g, 6a, b), whereas in sect. *Porphyrion*, they are located on the adaxial leaf lamina, albeit mostly close to the leaf margin (Figs. 1b–h, 2c–g, 4c–n, 5). Further investigation is required to determine the extent to which this characteristic represents a clear difference between *Ligulatae* and *Porphyrion* hydathodes, as the former are considered pseudomarginal or adaxial depending on the species. Only *Porphyrion* hydathodes are always adaxial on the leaf lamina (Zhang et al. 2015).

In any case, the hydathodes of both sections are similar in that they are sunken in deep pits with a few exceptions (see above). Additionally, their connection to the xylem involves a camptodromous leaf venation. This venation pattern is characteristic of lime-secreting species (Zhang et al. 2015), and already Unger (1861) correctly illustrated this pattern for the leaves of *S. crustata*. The presence of these pits with sunken hydathodes and the camptodromous venation of the leaves distinguish these species from all other saxifrages. The latter have water-secreting hydathodes that are flush with the adaxial leaf epidermis or marginal at the end of xylem strands. The leaves also have different venation types: mostly palinactinodromous (sections *Cotylea*, *Cymbalaria*, *Heterisia*, *Irregulares*, *Mesogyne*, and most *Saxifraga*); rarely acrodromous (section *Ciliatae*); or intermediate (sections *Gymnopera* and *Trachyphyllum*) (Fig. 7).

Therefore, we believe that Hypothesis 1 is correct. The sister group relationship between sections *Ligulatae* and *Gymnopera* is well established, based on molecular phylogenetic data and chromosomal findings, including the shared occurrence of the derived chromosome base number of *x* = 14 (Tkach et al. 2025), supporting their monophyly. Additionally, these sections have the unusual capability in saxifrages to produce naturally occurring intersectional hybrids. From an evolutionary perspective, we assume that a single origin and twofold loss of the lime-secreting hydathodes, namely in sections *Gymnopera* and *Trachyphyllum*, is most likely (Fig. 7). The strongest evidence supporting this scenario comes from the complexity of the lime secretion syndrome, which appears to consist of a coherent set of anatomical, morphological, and presumably biochemical and metabolic changes. Interestingly, *Gymnopera* and *Trachyphyllum* are the only sections displaying intermediate forms between camptodromous venation and other leaf venation patterns (Fig. 7). This is probably a reflection of their former possession of the lime secretion syndrome and the associated camptodromous leaf venations.

### Lime secretion as key innovation

The lime-secreting hydathodes could be a key innovation within *Saxifraga*, as was previously discussed (Conti et al. 1999; Ebersbach et al. 2017). Key innovations (Miller 1949) are newly acquired morphological, phenotypic, physiological, or ecological traits that lead to adaptive radiation through competitive advantages, ecomorphological divergence, and rich speciation. Examples within angiosperms include flora nectar spurs, resin channels, biochemical defenses against herbivory, and certain growth and fruit forms.

However, when comparing the sections based on species numbers, the section *Ligulatae* does not appear to be particularly species-rich, as it comprises only around 14 species. In contrast, the section *Porphyrion* is much more species-rich, with around 102 species. This could be understood as adaptive radiation resulting from an acquired key innovation (Fig. 7).

Nevertheless, the number of species within *Saxifraga* is significantly exceeded by that of the section *Ciliatae*, which has more than twice as many species (approximately 214). However, this section lacks lime-secreting hydathodes. Additionally, the likewise water-secreting section *Saxifraga*, with around 88 species, is not notably smaller than section *Porphyrion*, making it the third largest section of the genus. While many species in the section *Saxifraga* are calcicolous and grow on limestone or dolomite bedrock, none have lime-secreting hydathodes. Based solely on the numerical ratios of species richness in the aforementioned sections, the lime-secreting hydathodes of the sections *Ligulatae* and *Porphyrion* cannot be considered key innovations. Furthermore, upon closer inspection, an apparent increase in evolutionary diversification cannot be observed for section *Porphyrion* as a whole. At most, it can be attributed to one of its subsections, i.e. subsect. *Kabschia*, which contains approximately 93 species. However, the lime-secreting hydathodes cannot represent a key innovation for this subsection because these hydathodes are present throughout the section *Porphyrion*.

Therefore, we assume that the lime-secreting hydathodes of the sections *Ligulatae* and *Porphyrion* should not be considered a key innovation. This would require that this characteristic allowed these sections to exclusively colonize calcareous substrates in the *Saxifraga* phylogeny for the first time. However, since this is not the case, it would be of interest in future research to determine the physiological and functional significance of lime-secreting hydathodes for these plants. This would also explain why lime-secreting saxifrages often grow alongside water-secreting species on the same calcareous rocks in nature, as many field botanists know. Examples include *S. paniculata* and *S. exarata* (Webb and Gornall 1989; M.R., N.T., personal observations).

Nevertheless, it can be assumed that the lime-secreting ability plays an important ecological role in many *Porphyrion* species, which often inhabit extreme environments such as rocks and screes. This ability likely helps them cope with high electrolyte concentrations in soil water, particularly in situations of water shortage, which are to be expected in such locations. Many of these species also have a cushion-like growth form, which is likely another ecological adaptation to extreme thermal conditions caused by heat and cold, as well as dehydration due to frost in the absence of snow cover and the abrasive effect of wind in such exposed locations.

## Conclusion

Amphistomatic leaves are a unique adaptation in plant anatomy and physiology. The presence of stomata on both surfaces of the leaf enhances CO₂ uptake and photosynthetic efficiency in habitats with intense light and high daytime temperatures. While this arrangement increases the potential for water loss, the various patterns of stomata distribution among the studied saxifrage lineages suggest the presence of complementary structural and regulatory mechanisms that maintain hydration and thermal balance. These patterns seem to be species-specific. The variable distribution of stomata on the leaves highlights their adaptive value in response to various ecological pressures.

Additionally, the anatomy, physiology, ecology, and evolution of hydathodes in the Saxifragaceae family are fascinating. While the basic structure of vascular (xylem) termination, epithem tissue, and water pores is widely shared among *Saxifraga* and many other Saxifragaceae species, the morphology and function have been modified in some evolutionary lineages of the genus *Saxifraga*, particularly through lime secretion instead of the common water secretion, i.e., guttation of more or less xylem sap. These modifications likely reflect adaptations to calcareous, base-rich substrates with presumably high electrolyte concentrations in the soil water available to the numerous *Saxifraga* species inhabiting rocky areas. The modifications may have contributed to diversification by enabling new ecological niches. We believe that this lime secretion has only emerged once in the *Saxifraga* phylogeny. However, we do not consider it a key innovation because it is not unique to adaptive radiations. Such radiations have occurred in other *Saxifraga* groups without it.

Although the data is incomplete, the presence of lime-secreting hydathodes, along with other characteristics such as cushion formation and thick, succulent leaves, may have led to increased diversification within one of the lime-secreting saxifrage lineages, specifically the subsection *Kabschia*. Future integrative studies examining anatomy, physiology, molecular biology, and phylogeny in greater depth will make a decisive contribution to understanding how the unique lime-secreting hydathodes have shaped the adaptive and evolutionary development of saxifrages and their macroevolutionary role.

## Supplementary Information

Below is the link to the electronic supplementary material.

Supplementary file1 (XLSX 18 KB)

## Data Availability

All data supporting the findings of this study are available within the paper and its Supplementary Information.

## References

[CR1] Andrei M, Paraschivoiu RM (2008) Anatomical researches on the overground vegetative organs of *Saxifraga mutata* L. subsp. *demissa* (Schott and Kotschy) D.A. Webb and *Saxifraga paniculata* Miller. An Ştiinţ Univ Al I Cuza Iaşi Ser Nouă Sect 2a Biol Veg 54:5–14

[CR2] De Bary A (1877) Vergleichende Anatomie der Vegetationsorgane der Phanerogamen und Farne. In: Hofmeister W (ed) Handbuch der physiologischen Botanik. W. Engelmann, Leipzig

[CR3] Becraft PW (1999) Development of the leaf epidermis. Curr Top Dev Biol 45:1–40. 10.1016/S0070-2153(08)60313-0 10332602 10.1016/s0070-2153(08)60313-0

[CR4] Belin-DePoux M (1969) Contribution a l’étude des hydathodes. I. Remarques sur le type “à épithème” chez les Dicotylédones. Rev Gén Bot 76:631–657

[CR5] Bellenot C, Routaboul J-M, Laufs P, Noël LD (2022) Hydathodes. Curr Biol 32:R763–R764. 10.1016/j.cub.2022.06.014 35882191 10.1016/j.cub.2022.06.014

[CR6] Björn LO, Middleton BA, Germ M, Gaberščik A (2022) Ventilation systems in wetland plant species. Diversity Basel 14:517. 10.3390/d14070517

[CR7] Bothe H (2015) The lime-silicate question. Soil Biol Biochem 89:172–183. 10.1016/j.soilbio.2015.07.004

[CR8] Braun J (1913) Die Vegetationsverhältnisse der Schneestufe in den rätisch-lepontischen Alpen: ein Bild des Pflanzenlebens an seinen äussersten Grenzen. Zürcher und Furrer, Zürich. [Neue Denkschr Schweiz Naturf Ges 48]

[CR9] Carruthers T, Moerland MS, Ebersbach J, Favre A, Folk RA, Hawkins JA, Muellner-Riehl AN, Röser M, Soltis DE, Tkach N, Baker WJ, de Vos JM, Eiserhardt WL (2024) Repeated upslope biome shifts in *Saxifraga* during Late-Cenozoic climate cooling. Nat Commun 15:1100. 10.1038/s41467-024-45289-w 38321017 10.1038/s41467-024-45289-w PMC10847498

[CR10] Cerutti A, Jauneau A, Laufs P, Leonhardt N, Schattat MH, Berthomé R, Routaboul JM, Noël LD (2019) Mangroves in the leaves: anatomy, physiology, and immunity of epithemal hydathodes. Annu Rev Phytopathol 57:91–116. 10.1146/annurev-phyto-082718-100228 31100996 10.1146/annurev-phyto-082718-100228

[CR11] Conti E, Soltis DE, Hardig TM, Schneider J (1999) Phylogenetic relationships of the silver saxifrages (*Saxifraga* sect. *Ligulatae* Haworth): implications for the evolution of substrate specificity, life histories, and biogeography. Mol Phylogenet Evol 13:536–555. 10.1006/mpev.1999.0673 10620412 10.1006/mpev.1999.0673

[CR12] Cutler DF, Gregory M (1998) Anatomy of the dicotyledons. Saxifragales. Clarendon Press, Oxford

[CR13] Dingermann T, Kreis W, Nieber K, Rimpler H, Zündorf I (eds) (2016) Reinhard Pharmazeutische Biologie, 8th edn. Wissenschaftliche Verlagsgesellschaft, Stuttgart

[CR14] Drake PL, de Boer HJ, Schymanski SJ, Veneklaas EJ (2019) Two sides to every leaf: water and CO_2_ transport in hypostomatous and amphistomatous leaves. New Phytol 222:1179–1187. 10.1111/nph.15652 30570766 10.1111/nph.15652

[CR15] Ebersbach J, Schnitzler J, Favre A, Muellner-Riehl AN (2017) Evolutionary radiations in the species-rich mountain genus *Saxifraga* L. BMC Evol Biol 17:119. 10.1186/s12862-017-0967-2 28545386 10.1186/s12862-017-0967-2 PMC5445344

[CR16] Engler HGA, Irmscher E (1916) [„1919“] Saxifragaceae–*Saxifraga*. In: Engler HGA (ed) Das Pflanzenreich, vol 67. Engelmann, Leipzig, pp 1–448

[CR17] Engler HGA, Irmscher E (1919) Saxifragaceae*–Saxifraga*. In: Engler HGA (ed) Das Pflanzenreich, vol 69 (IV, 117). Engelmann, Leipzig, pp 449–709

[CR18] Evert R (2006) Esau’s plant anatomy meristems, cells, and tissues of the plant body: their structure, function, and development, 3rd edn. John Wiley and Sons, Hoboken, NJ

[CR19] Fahn A (1979) Secretory tissues in plants. Academic Press, London, New York

[CR20] Fehlauer T, Collin B, Angeletti B, Santaella C, Dentant C, Chaurand P, Levard CGC, Borschneck D, Rose J (2022a) Uptake patterns of critical metals in alpine plant species growing in an unimpaired natural site. Chemosphere 287:132315. 10.1016/j.chemosphere.2021.132315 34600011 10.1016/j.chemosphere.2021.132315

[CR21] Fehlauer T, Collin B, Angeletti B, Negahi MM, Dentant C, Chaurand P, Lallemand C, Levard C, Rose J (2022b) Multiscale imaging on *Saxifraga paniculata* provides new insights into yttrium uptake by plants. Sci Rep 12:18268. 10.1038/s41598-022-23107-x 36310318 10.1038/s41598-022-23107-x PMC9618566

[CR22] Galløe O (1910) 4. Saxifragaceae. 2. The biological leaf-anatomy of the arctic species of *Saxifraga*. In: Warming E (ed) (1908–1921) The structure and biology of arctic flowering plants. Meddel Grønland, pp 237–294

[CR23] Gardiner W (1881) The development of the water-glands in the leaf of *Saxifraga crustata*. Q J Microsc Sci 21:407–414. 10.1242/jcs.s2-21.83.407

[CR24] Guerrina M, Casazza G, Dagnino D, Macrì C, Roccotiello E, Minuto L (2020) Reproductive ecology of *Saxifraga florulenta*, a monocarpic perennial paleo-endemic of the Alps. Plant Biosyst 156:252–260. 10.1080/11263504.2020.1852328

[CR25] Haberlandt G (1894) Anatomisch-physiologische Untersuchungen über das tropische Laubblatt. II. Über wassersecernirende und -absorbirende Organe (I. Abhandlung). Sitzungsber Kaiserl Akad Wiss Wien Math-Naturwiss Cl Abt 1 103:489–538

[CR26] Hacker J, Neuner G (2006) Photosynthetic capacity and PSII efficiency of the evergreen alpine cushion plant *Saxifraga paniculata* during winter at different altitudes. Arct Antarct Alpine Res 38:198–205. 10.1657/1523-0430(2006)38[198:PCAPEO]2.0.CO;2

[CR27] Hayek A (1905) Monographische Studien über die Gattung *Saxifraga*. I. Die Sektion *Porphyrion* (Tausch.). Denkschr Kaiserl Akad Wiss Wien Math-Naturwiss Kl 77:611–709

[CR28] Hofmeister L (1939) Die Wasserpermeabilität der Zellen des Drüsenepithems von *Saxifraga*. Protoplasma 33:399–409. 10.1007/BF01788320

[CR29] Horný R, Webr KM, Byam-Grounds J (1986) Porophyllum saxifrages. J.S. Byam-Grounds, Stamford

[CR30] Islam MN, Kawasaki M (2015) Evaluation of calcium regulating roles of guttation and calcium oxalate crystals in leaf blades and petioles of hydroponically grown eddo. Plant Prod Sci 18:11–21. 10.1626/pps.18.11

[CR31] Jauneau A, Cerutti A, Auriac MC, Noël LD (2020) Anatomy of leaf apical hydathodes in four monocotyledon plants of economic and academic relevance. PLoS ONE 15(9):e0232566. 10.1371/journal.pone.0232566 32941421 10.1371/journal.pone.0232566 PMC7498026

[CR32] Jordan GJ, Carpenter RJ, Brodribb TJ (2014) Using fossil leaves as evidence for open vegetation. Palaeogeogr Palaeoclimatol Palaeoecol 395:168–175. 10.1016/j.palaeo.2013.12.035

[CR33] Jurzitza G (1987) Anatomie der Samenpflanzen. Georg Thieme Verlag, Stuttgart

[CR34] Kaplan K (1995) Saxifragaceae. In: Conert HJ, Jäger EJ, Kadereit JW, Schultze-Motel W, Wagenitz G, Weber HE (eds) Gustav Hegi. Illustrierte Flora von Mitteleuropa. Blackwell Wissenschafts-Verlag, Berlin, pp 130–229

[CR35] Köhlein F (1980) Saxifragen und andere Steinbrechgewächse. Ulmer, Stuttgart

[CR36] Kurt J (1930) Uber die Hydathoden der Saxifrageae. Beih Bot Centralbl 1. Abt 46:203–246

[CR37] Lazniewski W (1896) Beiträge zur Biologie der Alpenpflanzen. Flora 82:224–267

[CR38] Leistner E, Breckle S-W (2014) Pharmazeutische Biologie kompakt, 8th edn. Wissenschaftliche Verlagsgesellschaft, Stuttgart

[CR39] Liu Y, Liu H, Baastrup-Spohr L, Li Z, Li W, Pan J, Cao Y (2023) Allometric relationships between leaf and petiole traits across 31 floating-leaved plants reveal a different adaptation pattern from terrestrial plants. Ann Bot 131:545–552. 10.1093/aob/mcad007 36655615 10.1093/aob/mcad007 PMC10072084

[CR40] Liu C, Huang K, Zhao Y, Li Y, He N (2024) A continental-scale analysis reveals the latitudinal gradient of stomatal density across amphistomatous species: Evolutionary history vs. present-day environment. Ann Bot 134:877–886. 10.1093/aob/mcae135 39136155 10.1093/aob/mcae135 PMC11639198

[CR41] McGregor M (2008) Saxifrages, a definitive guide to the 2000 species, hybrids and cultivars. Timber Press, Portland, OR

[CR42] Metcalfe CR, Chalk L (1950) Anatomy of the dicotyledons, vol 1. Clarendon Press, Oxford

[CR43] Metcalfe CR, Chalk L (1979) Anatomy of the dicotyledons, 2nd edn. Clarendon Press, Oxford

[CR44] Michavila S, Encina A, Frey C, Álvarez R (2021) Histological description of *Saxifraga paniculata* leaves with special focus on structures that release CaCO_3_. Plant Biosyst 156:497–505. 10.1080/11263504.2021.1887954

[CR45] Miller AH (1949) Some ecologic and morphologic considerations in the evolution of higher taxonomic categories. In: Mayr E, Schulz E (eds) Ornithologie als Biologische Wissenschaft. Carl Winter, Heidelberg, pp 84–88

[CR46] Moreau F (1971) Apport des caractères stomatiques à la taxinomie et à la phylogénie des Saxifragées. Bull Soc Bot Fr 118:381–427. 10.1080/00378941.1971.10838914

[CR47] Moreau F (1984) Contribution phytodermologique à la systématique des saxifragacées sensu stricto et des crassulacées. Rev Cytol Biol Végét, Le Botaniste 7:31–92

[CR48] Mott KA, Gibson AC, O’Leary JW (1982) The adaptive significance of amphistomatic leaves. Plant Cell Environ 5:455–460

[CR49] Muir C, Lim W, Wan D (2025) Plasticity and adaptation to high light intensity amplify the advantage of amphistomatous leaves. Preprint: 10.1101/2025.08.19.671015

[CR50] Napp-Zinn K (1974) Handbuch der Pflanzenanatomie. Anatomie des Blattes 2 Blattanatomie der Angiospermen. Borntraeger, Berlin

[CR51] Neuner G, Braun V, Buchner O, Taschler D (1999) Leaf rosette closure in the alpine rock species *Saxifraga paniculata* Mill.: significance for survival of drought and heat under high irradiation. Plant Cell Environ 22:1539–1548. 10.1046/j.1365-3040.1999.00508.x

[CR52] Parkhurst DF (1978) The adaptive significance of stomatal occurrence on one or both surfaces of leaves. J Ecol 66:367–383. 10.2307/2259152

[CR53] Pillitteri LJ, Bogenschutz NL, Torii KU (2008) The bHLH protein, MUTE, controls differentiation of stomata and the hydathode pore in *Arabidopsis*. Plant Cell Physiol 49:934–943. 10.1093/pcp/pcn067 18450784 10.1093/pcp/pcn067

[CR54] Pillitteri LJ, Dong L (2013) Stomatal development in *Arabidopsis*. In: The American Society of Plant Biologists (ed) The *Arabidopsis* Book 11:e0162. 10.1199/tab.0162 10.1199/tab.0162 PMC3711358 23864836

[CR55] Routaboul J-M, Bellenot C, Olympio A, Clément G, Citerne S, Remblière C, Charvin M, Franke L, Chiarenza S, Vasselon D, Jardinaud M-F, Carrère S, Nussaume L, Laufs P, Leonhardt N, Navarro L, Schattat M, Noël LD (2024) *Arabidopsis* hydathodes are sites of auxin accumulation and nutrient scavenging. Plant J 120(3):857–871. 10.1111/tpj.17014 39254742 10.1111/tpj.17014

[CR56] Schmidt H (1930) Zur Funktion der Hydathoden von *Saxifraga*. Planta 10:314–344. 10.1007/BF01911458

[CR57] Singh S (2014) Guttation: new insights into agricultural implications. In: Sparks DL (ed) Advances in agronomy, vol 128. Elsevier, San Diego, CA, pp 97–135. 10.1016/B978-0-12-802139-2.00003-2

[CR58] Taiz L, Møller IM, Murphy A, Zeiger E (2023) Plant physiology and development, 7th edn. Oxford University Press, Oxford

[CR59] Takeda F, Wisniewski ME, Glenn D (1991) Occlusion of water pores prevents guttation in older strawberry leaves. J Am Soc Hortic Sci 116:1122–1125. 10.21273/JASHS.116.6.1122

[CR60] Thiers BM (updated continuously) Index herbariorum. Available at: https://sweetgum.nybg.org/science/ih/

[CR61] Tkach N, Röser M, Miehe G, Muellner-Riehl AN, Ebersbach J, Favre A, Hoffmann MH (2015) Molecular phylogenetics, morphology and a revised classification of the complex genus *Saxifraga* (Saxifragaceae). Taxon 64:1159–1187. 10.12705/646.4

[CR62] Tkach N, Spielau S, Horák D, Röser M (2025) Genome sizes and chromosomal restructuring in the evolution of *Saxifraga* and other Saxifragaceae. Preprint: 10.21203/rs.3.rs-8115725/v1

[CR63] Torii KU (2021) Stomatal development in the context of epidermal tissues. An Bot 128:137–148. 10.1093/aob/mcab052 10.1093/aob/mcab052 PMC8324025 33877316

[CR64] Troll W (1939) Vergleichende Morphologie der höheren Pflanzen, Bd. 1, Teil 2. Morphologie des Blattes. Gebrüder Borntraeger, Berlin

[CR65] Unger F (1836) Ueber den Einfluss des Bodens auf die Vertheilung der Gewächse: nachgewiesen in der Vegetation des nordöstlichen Tirol’s. Rohrmann and Schweigerd, Wien. 10.5962/bhl.title.51507

[CR66] Unger F (1861) Beiträge zur Physiologie der Pflanzen (Fortsetzung). VIII. Über die kalkausscheidenden Organe der *Saxifraga crustata* Vest. Sitzungsber Math-Naturwiss Cl Kaiserl Akad Wiss Wien 2 Abt 43:519–527

[CR67] Van Cotthem WRJ (1970) A classification of stomatal types. Bot J Linn Soc 63:235–246. 10.1111/j.1095-8339.1970.tb02321.x

[CR68] Volkens G (1883) III. Über Wasserausscheidung in liquider Form an den Blättern höherer Pflanzen. Jahrb Königl Bot Gart Bot Mus Berlin 2:166–209

[CR69] Wagner A (1892) Zur Kenntniss des Blattbaues der Alpenpflanzen und dessen biologische Bedeutung. Sitzungsber Kaiserl Akad Wiss, Wien, Math-Naturwiss Cl 101:487–548

[CR70] Waldner M (1878) Die Kalkdrüsen der Saxifragen. Mitt Naturwiss Vereins Steiermark 14:25–33

[CR71] Webb DA, Gornall RJ (1989) Saxifrages of Europe: with notes on African, American and some Asiatic species. C. Helm, London

[CR72] Wightman R, Wallis S, Aston P (2017) Hydathode pit development in the alpine plant *Saxifraga cochlearis*. Flora 233:99–108. 10.1016/j.flora.2017.05.018

[CR73] Wightman R, Wallis S, Aston P (2018) Leaf margin organisation and the existence of vaterite-producing hydathodes in the alpine plant *Saxifraga scardica*. Flora 241:27–34. 10.1016/j.flora.2018.02.006

[CR74] Zhang Z, Xia N, Gornall RJ (2015) Leaf venation patterns in the genus *Saxifraga* (Saxifragaceae). Phytotaxa 221:123–136. 10.11646/phytotaxa.221.2.2

[CR75] Zhmylev PY, Kovalenko MA (2023) Kamnelomki Rossii [Saxifrages of Russia]. State University “Dubna,” Dubna

